# Age-related differences in patient-reported quality of care among adult German patients with bronchial asthma: a cross-sectional study

**DOI:** 10.1038/s41533-026-00492-8

**Published:** 2026-02-23

**Authors:** Anu Wank, Merle Fresemann, Lukas Schöner, Janis Nikkhah, Laura Wittich

**Affiliations:** Department of Health Care Management, Technical University of Berlin, Berlin, Germany

**Keywords:** Diseases, Health care, Medical research

## Abstract

Limited evidence exists on age-related differences in health-related quality of life (HRQoL) and patient-reported outcome and experience measures (PROMs/PREMs) among asthma patients. This study analysed data from 765 adults in the German PROMchronic trial, comparing generic HRQoL, asthma-control, and PREMs across age groups (18–44, 45–64, 65–74, ≥75 years), with analyses stratified by gender. Older adults, particularly women aged 65–74 years, reported slightly higher HRQoL (p = 0.004, η² = 0.017), and ≥75 aged reported better asthma control scores (p = 0.012, Cliffs Delta = 0.261). Categorical asthma control and most PREM domains did not differ significantly. Organisational aspects of care were rated more favourably by adults aged 65–74 years (p = 0.040, Cramér’s V = 0.104), especially women. Age-related differences in PROMs and PREMs were small. These findings suggest subtle but relevant patterns in patient-reported quality of asthma care and support age-sensitive, patient-centred approaches in primary care.

## Introduction

Asthma is one of the most prevalent chronic respiratory diseases worldwide^1^, frequently accompanied by comorbid conditions such as gastroesophageal reflux, allergies, or chronic obstructive pulmonary disease (COPD)^2^. Appropriate treatment enables most patients to achieve symptom control and a life expectancy comparable to that of the general^3^; whereas untreated asthma can be fatal^4^.

The primary goal of asthma management is sustained disease control, typically defined by preserved lung function, infrequent symptoms, and minimal disruption to daily^5^, rather than cure^3^. In Germany, structured disease management programmes (DMPs) promote coordinated, evidence-based care to reduce complications^6^. As of 2023, over one million asthma patients were enrolled in a DMP^7^, with 84.1% classified as having controlled asthma^8^. Based on clinical indicators, asthma care therefore appears effective in achieving disease control.

However, quality assessments continue to rely mainly on physiological indicators such as spirometry, while patients’ subjective experiences and perceived outcomes receive comparatively little attention^2^. In a postal survey of 2568 statutorily health-insured German adults with asthma, including both DMP participants and non-participants, two-thirds (66.1%) reported daily or intermittent asthma-related limitations such as frequent daytime or nocturnal symptoms^9^. This indicates that self-reported burden remains substantial despite high clinical control rates documented within DMP quality reporting^10^.

Patient-reported outcome measures (PROMs) and patient-reported experience measures (PREMs) provide complementary insights into patients’ health status and care experiences^11,12^. PROMs capture health-related quality of life (HRQoL) and disease-specific symptoms, while PREMs assess experiences with the healthcare system. Despite their value for person-centred evaluation, PROMs and PREMs are not integrated into routine asthma care in Germany^13^.

Importantly, PROM findings vary across patient subgroups, with age being one factor associated with how patients experience and report their quality of care^14^. Yet, evidence on age-related variation in HRQoL among patients with asthma remains limited and inconsistent. A cross-sectional study of German adults found that both physical and mental HRQoL scores were significantly lower among women, older adults, and those reporting insufficient asthma control or symptoms of anxiety or depression^15^. Longitudinal evidence from a two-year Dutch cohort similarly showed that older age predicted a decline in HRQoL, yet this decline was only weakly related to changes in lung function, suggesting that HRQoL reflects aspects of disease burden not captured by physiological measures^16^. In the population-based European Community Respiratory Health Survey (ECRHS II), which included 837 patients with asthma, both disease-specific and generic scores were lower than population norms; the physical, but not the mental component of HRQoL decreased with greater asthma severity, more frequent attacks, nocturnal symptoms, and recent hospitalizations, although age was included only as a covariate rather than examined as a predictor^17^. A subsequent Nordic follow-up of the ECRHS cohort comprising 2,270 adults aged 29–55 years from Sweden, Iceland, and Norway reported that higher age and body mass index were associated with poorer physical health scores; however, older age correlated with better mental health scores after adjustment. HRQoL was also independently related to anxiety, depression, and insomnia, indicating that psychological and behavioural factors contributed to the perceived disease burden beyond clinical control^18^.

Taken together, these studies indicate that HRQoL is consistently lower among asthma patients relative to the general population. At the same time, age-related differences are inconsistent and domain-specific, typically showing lower physical but more stable or even better mental well-being in older adults. This evidence highlights the need for research explicitly examining age-related differences in patient-reported outcomes and experiences in asthma care, and the potential of PROMs to capture such age-sensitive dimensions of patient-centred quality of care. The aim of the present study is to investigate differences across age-groups in generic HRQoL, disease-specific asthma control, and patient-reported experiences of care among adults with asthma in Germany.

## Methods

### Study design and participants

This analysis was based on data from the *PROMchronic* trial, a prospective cohort study conducted between October 2023 and September 2024. *PROMchronic* included adult patients diagnosed with one of four chronic diseases: bronchial asthma, COPD, coronary artery disease (CAD), or type 1 or type 2 diabetes^19^.

In October 2023, a stratified sample of 200,338 adults was drawn from the BARMER-insured population (approximately 50,000 per disease, combining type 1 and type 2 diabetes). Eligibility criteria included age over 18 years and a confirmed diagnosis based on administrative claims data, defined as having an ICD-10-GM diagnosis code for the respective chronic disease recorded with repeated occurrences in at least two distinct treatment cases in 2021 (M2B-criterion^20,21^). Patients enrolled in more than one disease management programme (DMP) were excluded. For the present analysis, only patients with a diagnosis of bronchial asthma were included.

Participants completed digital health surveys every three months for one year. Demographic data were collected in the first survey. The present analysis includes data from the initial survey period only.

### Measures

#### Patient-reported outcome measures (PROMs)

PROMs were used to assess the subjective health status. Two instruments were applied:

The generic *Patient Reported Outcomes Measurement Information System (PROMIS)- Preference Score* (PROPr)^22^ is a preference-based index aggregating seven PROMIS domains into a single score, ranging from 1.0 (full health) to −0.022 (worse than death), with a value of 0 representing death. Higher PROPr indicates better health-related quality of life.

The disease-specific *Asthma Impairment and Risk Questionnaire* (AIRQ)^23^ comprises 10 items measuring asthma control. Scores range from 0 to 10, with lower values indicating well-controlled asthma (0–1: well controlled; 2–4: not well controlled; 5–10: very poorly controlled).

Both instruments are validated and available in German.

#### Patient-reported experience measures (PREMs)

Care experience was assessed using the *Responsiveness* questionnaire^24^. This PREM captures patients’ evaluations of their most recent outpatient consultation with a general practitioner or specialist in the past 12 months. Items are rated on a five-point Likert scale and were categorised for analysis into five groups: very good, good, moderate, bad, and very bad. Responsiveness includes two domains:

- *Organisational domain*: structural and procedural aspects such as waiting times and provider choice.
- *Interpersonal domain*: interactional aspects such as respect, autonomy, and communication^12^.

The instrument is validated for use with patients with chronic diseases and is available in German.

#### Independent variable and covariates

Age was the primary independent variable, measured in years and categorised into four groups: 18–44, 45–64, 65–74, and ≥75 years. This categorisation reflects key life-course stages and the epidemiology of chronic conditions^25^ while ensuring sufficient case numbers in each category.

Sociodemographic covariates included gender, body mass index (BMI), household income, education, employment status, and household size. These were obtained from the first survey.

### Statistical analyses

All analyses were performed in R (version 4.4.2). The significance level was set at α = 0.05.

Descriptive statistics were reported as means (standard deviations) or medians (interquartile ranges) for continuous variables, and frequencies and percentages for categorical variables.

Group comparisons were conducted to examine differences in PROM and PREM results across age groups. For *continuous PROMs* (PROPr and AIRQ), one-way analysis of variance (ANOVA) was applied when assumptions of normality (Shapiro-Wilk test, Q-Q plots) and homogeneity of variance (Levene’s test) were met^26^. If assumptions were violated, the non-parametric Kruskal-Wallis test was used^27^.

Post-hoc comparisons were performed with Tukey’s test for ANOVA^26^ and Dunn’s test for significant Kruskal-Wallis results^28^. Effect sizes were reported using eta-squared (η²) for ANOVA and Kruskal-Wallis test (small: 0.01, medium: 0.06, large: 0.14)^29^, Cohens d for Tukey’s tests (small: <0.2, medium: <0.5, large: <0.8), and Cliff’s Delta for Dunn’s tests (small: |0.15|, medium: |0.33|, large: |0.47|)^30^.

Both the continuous AIRQ total score and the derived categorical asthma control levels were analysed. The constant score captures fine-grained differences between patients and allows precise quantitative comparisons, whereas the categorical control levels translate these numerical data into clinically relevant categories that are easily interpretable^23^.

For *categorical outcomes* (asthma control levels and PREM scores), group differences were assessed using chi-squared tests for homogeneity. If expected cell frequencies fell below five, Fisher’s exact test was used instead^31^. Significant results were compared pairwise using the same initial test. Effect sizes were calculated using Cramér’s V or Phi coefficient for 2 × 2 tables (small: 0.1, medium: 0.3, large: 0.5)^29^. PREM responses were dichotomised into ‘very good/good’ and ‘moderate/bad/very bad’^12^ due to small sample sizes in some categories.

Analyses were stratified by gender. Due to low counts in the ‘diverse’ category, gender analyses were restricted to men and women. Analyses were conducted as descriptive, unadjusted comparisons across age groups. Adjustment for confounding was not performed because several clinically relevant variables for asthma outcomes (e.g., comorbidity burden, asthma severity/phenotype, and DMP enrolment) were not available in sufficient detail in the dataset used for this manuscript.

A large language model–based assistant (ChatGPT, developed by OpenAI) was used solely for language editing and consistency checking. All analyses, results, and interpretations were developed and verified independently by the authors.

#### Multiple comparisons and missing data

To control the alpha error rate, p-values from pairwise comparisons were adjusted using the Holm correction^32^. Missing data (<5% for PROM and PREM scores) were handled using complete-case analysis, which was deemed appropriate given the low proportion of missing values^33^.

#### Sensitivity analyses

Sensitivity analyses were conducted using a dichotomised age variable (<65 years vs. ≥65 years^34^) to test the robustness of findings against the primary four-group categorisation. The age threshold of 65 years corresponds approximately to the statutory retirement age in Germany and was therefore considered a meaningful cut-off point^35^.

## Results

### Patient characteristics

**Fig. 1:**
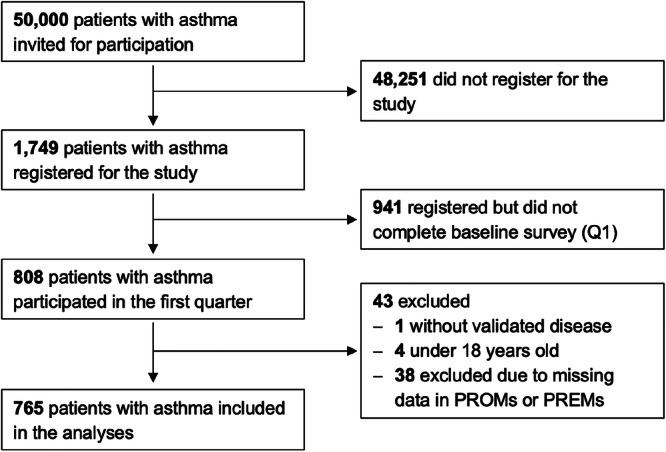
Participant flow diagram of asthma patients included in the analyses. Demographic data were collected in the baseline survey (Q1). The present analysis includes data from the initial survey period only.

A total of 765 patients with asthma were included, as shown in Fig. 1.

The largest age group was 45–64 years (n = 447), while those ≥75 years were the smallest (n = 49). Table 1 presents descriptive characteristics by age group. Across all groups, the majority were women (60.7%–77.0%).

**Table 1:** Sociodemographic characteristics stratified by age group.

	18–44 (n = 134)	45–64 (n = 447)	65–74 (n = 135)	≥ 75 (n = 49)
**Age**				
Mean (SD)	36.14 (6.85)	55.40 (5.27)	68.82 (2.77)	78.53 (3.16)
**Gender** n (%)				
Women	98 (73.1)	344 (77.0)	82 (60.7)	32 (65.3)
Men	35 (26.1)	102 (22.8)	53 (39.3)	17 (34.7)
Diverse	1 (0.8)	1 (0.2)	0 (0.0)	0 (0.0)
**BMI** n (%)				
Underweight	4 (3.0)	7 (1.6)	1 (0.7)	1 (2.0)
Normal weight	44 (32.84)	129 (28.9)	47 (34.8)	17 (34.7)
Overweight	33 (24.63)	147 (32.9)	53 (39.3)	20 (40.8)
Obesity	50 (37.31)	157 (35.1)	32 (23.7)	9 (18.4)
Missing	3 (2.24)	7 (1.6)	2 (1.5)	2 (4.1)
**Income (in Euros)**^a^ n (%)				
Up to 1,499	10 (7.5)	27 (6.0)	12 (8.9)	3 (6.1)
1,500 to 2,499	28 (20.9)	68 (15.2)	29 (21.5)	12 (24.5)
2,500 to 4,499	28 (20.9)	121 (27.1)	40 (29.6)	10 (20.4)
4,500 to 5,499	18 (13.4)	50 (11.2)	10 (7.4)	2 (4.9)
5,500 or more	12 (9.0)	34 (7.6)	7 (5.2)	1 (2.0)
I don’t know or prefer not to say	35 (26.1)	141 (31.5)	35 (25.9)	19 (38.8)
Missing	3 (2.2)	6 (1.3)	2 (1.5)	2 (4.1)
**Education**^b^ n (%)				
Low Education	7 (5.2)	48 (10.7)	26 (19.3)	10 (20.4)
Medium Education	80 (59.7)	305 (68.2)	73 (54.1)	28 (57.1)
High Education	41 (30.6)	84 (18.8)	30 (22.2)	7 (14.3)
Other	3 (2.2)	4 (0.9)	4 (3.0)	2 (4.1)
Missing	3 (2.2)	6 (1.3)	2 (1.5)	2 (4.1)
**Work situation** n (%)				
Not working	7 (5.2)	12 (2.7)	1 (0.7)	0 (0.0)
Not working because of disease	7 (5.2)	15 (3.4)	2 (1.5)	0 (0.0)
Retired	3 (2.2)	54 (12.1)	106 (78.5)	47 (95.9)
Working	102 (76.1)	359 (80.3)	24 (17.8)	0 (0.0)
Missing	15 (11.2)	7 (1.6)	2 (1.5)	2 (4.1)
**Persons per household** n (%)				
1	33 (24.6)	88 (19.7)	27 (20.0)	11 (22. 5)
2	43 (32.1)	235 (52.6)	97 (71.9)	35 (71.4)
3	27 (20.2)	69 (15.4)	9 (6.7)	1 (2.0)
4 or more	28 (20.9)	49 (11.0)	0 (0.0)	0 (0.0)
Missing	3 (2.2)	6 (1.3)	2 (1.5)	2 (4.1)

*SD* standard deviation, *BMI* body mass index.

^a^Average monthly net income of the household.

^b^Classified according to the International Standard Classification of Education (ISCED)^59^.

Most patients aged 18–44 years (76.1%) and 45–64 years (80.3%) were employed, while the majority of patients aged 65–74 years (78.5%) and ≥75 years (95.9%) were retired. The proportion of patients unable to work due to illness ranged from 0.0% to 5.2%, with the lowest values observed in older age groups.

### PROMs by age group

Summary statistics for the generic PROM PROPr and the disease-specific PROM AIRQ are shown in Table 2.

**Table 2:** Summary statistics for PROPr, AIRQ, and asthma control stratified by age.

PROM^*a*^	18–44 (n = 134)	45–64 (n = 447)	65–74 (n = 135)	≥ 75 (n = 49)	Test (Statistic, p, Effect size)
**PROPr**					ANOVA, F(3, 761) = 4.494, p = 0.004, η² = 0.017 65–74 vs. 18–44 Tukey’s test, p = 0.046, Cohen’s d = −0.317 65–74 vs. 45–64 Tukey’s test, p = 0.004, Cohen’s d = −0.326
Mean (SD)	0.38 (0.20)	0.38 (0.21)	0.45 (0.22)	0.43 (0.19)	
Median (IQR)	0.38 (0.242 - 0.512)	0.36 (0.220 - 0.520)	0.44 (0.302 - 0.602)	0.44 (0.318 - 0.531)	
**AIRQ**					Kruskal-Wallis test, χ^2^(3) = 12.775, p = 0.005, η² = 0.013 45–64 vs. ≥75 Dunn’s test, p = 0.012, Cliff’s Delta = 0.261
Mean (SD)	2.26 (2.24)	2.67 (2.49)	2.17 (2.34)	1.61 (2.2)	
Median (IQR)	2 (0–4)	2 (0–4)	1 (0–4)	1 (0–2)	
**Asthma control** n (%)					Fisher’s exact test, p = 0.063, Cramér’s V = 0.089
Well-controlled	61 (45.5)	185 (41.4)	68 (50.4)	29 (59.2)	
Not well-controlled	51 (38.1)	155 (34.7)	44 (32.6)	15 (30.6)	
Very poorly controlled	22 (16.4)	107 (23.9)	23 (17.0)	5 (10.2)	

*PROM* Patient-reported outcome measure, *ANOVA* Analysis of Variance, *AIRQ* Asthma Impairment and Risk Questionnaire, *PROPr* PROMIS-Preference Score, *SD* standard deviation, *IQR* interquartile range.

^a^The score ranges for the PROMs are −0.022 to 1 for the PROPr, with higher scores indicating better health, and 0 to 10 for the AIRQ, with higher scores indicating poorer asthma control (0 to 1: Well controlled, 2 to 4: Not well controlled, 5 to 10: Very poorly controlled).

#### Generic health-related quality of life (PROPr)

In the age groups of 18–44 and 45–64 years, mean PROPr scores were 0.38 (SD = 0.20) and 0.38 (SD = 0.21), respectively, compared with 0.45 (SD = 0.22) in the age groups of 65–74 years and 0.43 (SD = 0.19) in the age group of ≥75 years. The overall difference across age groups was statistically significant (ANOVA, F(3, 761) = 4.494, p = 0.004, η² = 0.017). Post-hoc Tukey’s tests showed that patients aged 65–74 years reported significantly better HRQoL than those aged 18–44 (p = 0.046, Cohen’s d = −0.317) and 45–64 (p = 0.004, Cohen’s d = −0.326), both with small effect sizes.

Among women, a similar pattern was observed (F(3, 552) = 3.622, p = 0.013, η² = 0.019). As in the overall analysis, women aged 65–74 years reported higher PROPr scores than those aged 18–44 years (p = 0.032, Cohen’s d = −0.415) and 45–64 years (p = 0.012, Cohen’s d = −0.377), again with small effect sizes. Among men, no significant differences were observed (see Supplementary Table 1).

#### Disease-specific asthma control (AIRQ)

**Fig. 2:**
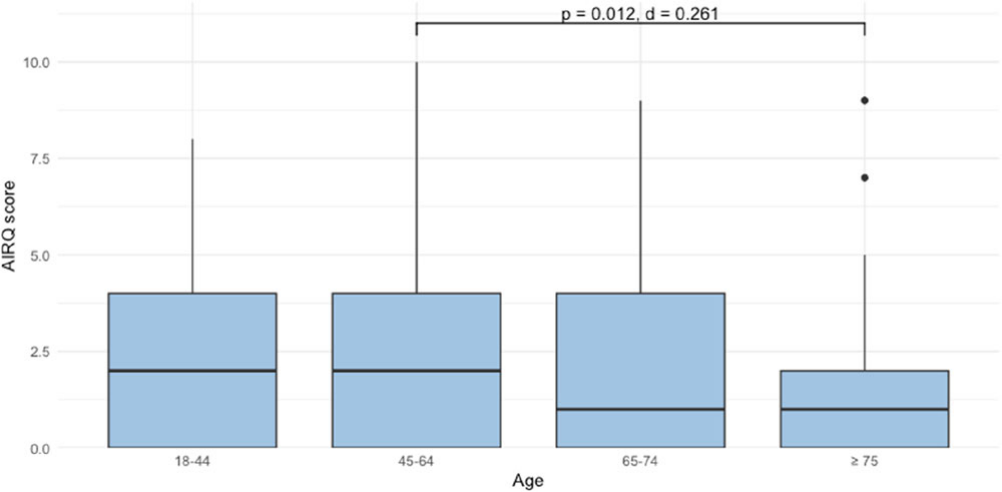
Comparison of AIRQ scores across age groups. Box plots show the distribution of AIRQ scores by age group. Significant differences between groups are indicated by bars, with corresponding adjusted p-values (Dunn’s test) and effect sizes (Cliff’s delta). Boxplot elements: Box limits = Q1–Q3, median = centre line, whiskers = 1.5x interquartile range, points = outliers.

Median AIRQ scores were highest in the 45–64 years age group and lowest in the ≥75 years group (see Table 2). Overall differences across age groups were significant (Kruskal-Wallis test, χ^2^(3) = 12.775, p = 0.005, η² = 0.013). Post-hoc Dunn’s tests indicated better control in patients aged ≥75 compared with those aged 45–64 years, with a small effect size (p = 0.012, Cliff’s Delta = 0.261) (see Fig. 2).

Among women, group differences were significant (p = 0.045), but no pairwise comparisons reached significance. Among men, no significant differences were observed (see Supplementary Table 1).

When AIRQ was categorised into control levels (well controlled, not well controlled, very poorly controlled), more than 40% of patients across all groups had well-controlled asthma, with the highest proportion in the ≥75 years group (59.2%). This group also had the lowest proportion of patients with very poorly controlled asthma (10.2%), compared to 16.4% in the 18–44 years group and 23.9% in the 45–64 years group. The proportion of patients classified as not well-controlled remained relatively stable across all age groups, ranging from 30.6% (≥75) to 38.1% (18–44).

**Fig. 3:**
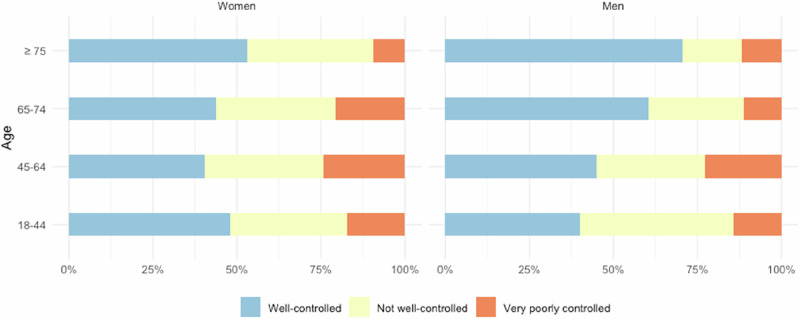
Asthma control levels stratified by age and gender. Proportion of patients with well-controlled, not well-controlled, and very poorly controlled asthma.

Differences across groups neither reached statistical significance nor were differences observed in gender-stratified analyses (see Fig. 3).

### PREMs by age group

Across all age groups, most patients rated their care experience as ‘very good/good’, ranging from 86.4% (45–64 years) to 92.5% (65–74 years). Interpersonal aspects of care were consistently rated more positively than organisational aspects. Table 3 shows the frequencies and percentages of the PREM Responsiveness categories, alongside the test results.

**Table 3:** Summary statistics for PREM Responsiveness stratified by age.

PREM^a^	18–44 (n = 134)	45–64 (n = 447)	65–74 (n = 135)	≥ 75 (n = 49)	Test (Statistic, p, Effect size)
**Responsiveness** n (%)					Fisher’s exact test, p = 0.250, Cramer’s V = 0.075
Moderate/bad/very bad	14 (10.5)	61 (13.7)	10 (7.5)	5 (10.2)	
Very good/good	120 (89.6)	386 (86.4)	124 (92.5)	44 (89.8)	
Not applicable	0	0	1	0	
**Responsiveness – Organisational domain** n (%)					Chi-squared test, χ^2^(3) = 8.298, p = 0.040, Cramér’s V = 0.104 65–74 vs. 45–64, Chi-squared test, χ^2^(1) = 7.534, p = 0.047, Phi = 0.110
Moderate/bad/very bad	38 (28.4)	132 (29.5)	24 (17.9)	10 (20.4)	
Very good/good	96 (71.6)	315 (70.5)	110 (82.1)	39 (79.6)	
Not applicable	0	0	1	0	
**Responsiveness – Interpersonal domain** n (%)					Fisher’s exact test, p = 0.765, Cramér’s V = 0.042
Moderate/bad/very bad	9 (6.7)	41 (9.2)	9 (6.7)	4 (8.2)	
Very good/good	125 (93.3)	406 (90.8)	125 (93.3)	45 (91.8)	
Not applicable	0	0	1	0	

*PREM* Patient-reported experience measure.

^a^PREM responses were dichotomized into ‘very good/good’ and ‘moderate/bad/very bad’.

**Fig. 4:**
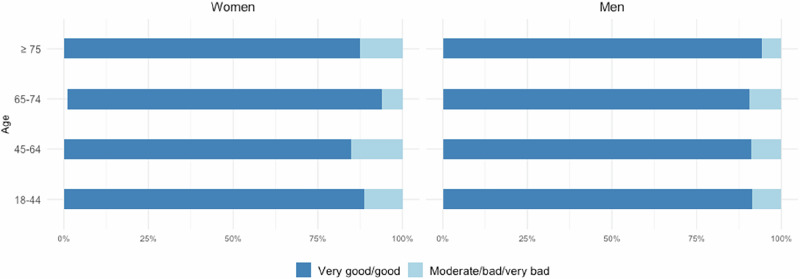
PREM Responsiveness ratings stratified by age and gender. Distributions of patients with ‘very good/good’ and ‘moderate/bad/very bad’ experience. See Supplementary Table 2 for detailed statistics.

No overall age-group differences were found in Responsiveness ratings. Gender-stratified analyses likewise revealed no differences (see Fig. 4).

**Fig. 5:**
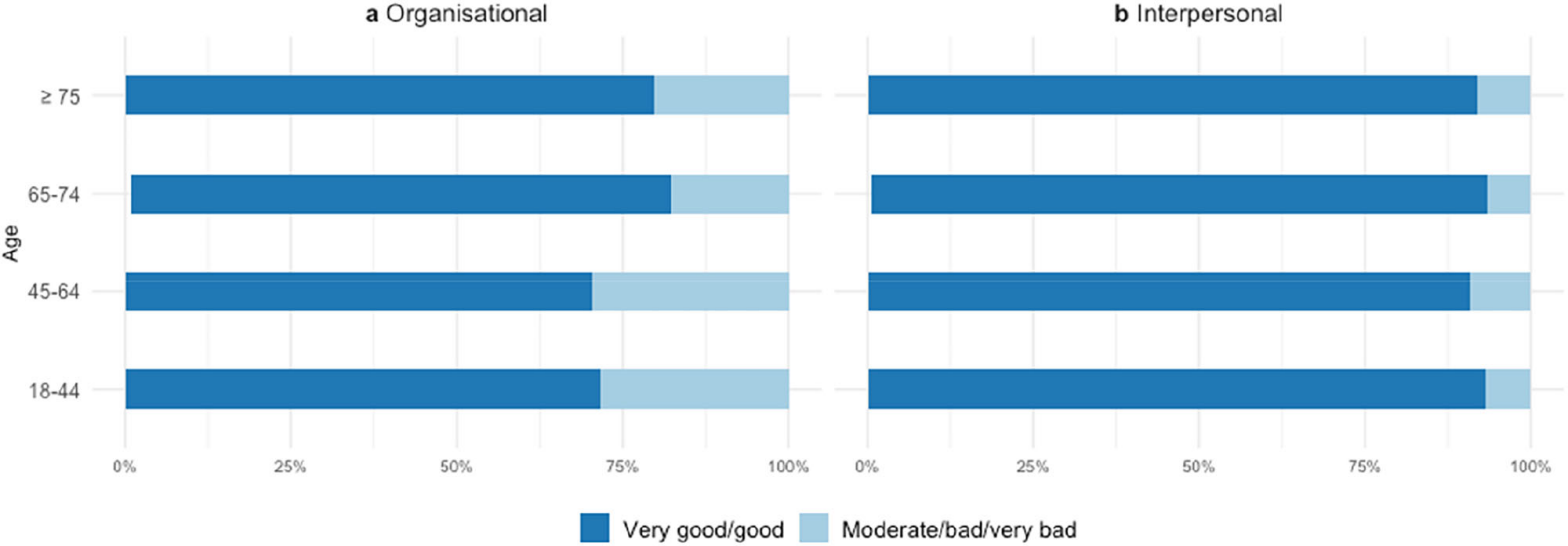
PREM Responsiveness ratings stratified by age in organisational and interpersonal domains. Distributions of patients with 'very good/good' and 'moderate/bad/very bad' experience across age groups. Panel **a** shows organisational domain ratings, reflecting structural and procedural factors. Panel **b** shows interpersonal domain ratings, reflecting interactions between patients and healthcare providers. See Supplementary Table 2 for detailed statistics.

In the 45–64 years age group, 29.5% rated their *organisational experience* as ‘moderate/bad/very bad’, while 70.5% rated it as ‘very good/good’. In comparison, the 65–74 years age group reported more positive experiences overall, with 17.9% providing a negative rating and 82.1% reporting a positive experience (*see* Fig. 5).

Group differences were significant (Chi-squared test, χ^2^(3) = 8.298, p = 0.040, Cramér’s V = 0.104), with a small effect size. Post-hoc comparison revealed higher positive ratings among 65-74-year-olds than among 45-64-year-olds (p = 0.047, Phi = 0.110), with a small effect size.

Among women, organisational experiences improved with age (p = 0.009), with significant differences between 65–74 years compared to 18–44 years (p = 0.027, Phi = 0.208) and between 65–74 years and 45–64 years (p = 0.006, Phi = 0.161). Among men, no significant differences were observed (see Supplementary Table 2).

Ratings in the interpersonal domain were consistently high across all groups, with over 90% of patients in each age group reporting very good or good experiences. No significant differences were observed by age group or gender.

### Sensitivity analyses

Group sizes were unequal, particularly in the ≥75-year group. To assess robustness against the primary four-group categorisation, we performed sensitivity analyses using a dichotomised age variable ( < 65 vs ≥65 years), which confirmed the main findings as for PROPr (ANOVA, F(1, 763) = 13.27, p = 0.001, η² = 0.017) and PREM Responsiveness (Chi-squared test, χ^2^(1) = 2.537, p = 0.111, Cramér’s V = 0.062). However, some nuances were lost: While the four-group analysis identified improvements in AIRQ concentrated among ≥75-year-olds, the dichotomised analysis suggested broader benefits among all patients ≥65 (Kruskal-Wallis test, χ^2^(1) = 8.337, p = 0.004, η² = 0.010), particularly men (p = 0.020, η² = 0.006). Patients ≥65 years were more likely to report well-controlled asthma and less likely to report very poorly controlled asthma compared with younger patients (Chi-squared test, χ^2^(2) = 7.143, p = 0.028, Cramér’s V = 0.028). Stratified analyses showed no significant differences among women, whereas older men reported substantially better asthma control than men aged <65 (p = 0.031, Cramér’s V = 0.183). In contrast, the four-group approach did not reveal significant age-related differences.

## Discussion

This cross-sectional analysis of 765 adults with asthma found small age- and gender-related differences in patient-reported quality of care. HRQoL (PROPr) was higher in patients aged 65–74 years compared with those aged 18–44 years and 45–64 years, with small effect sizes. This age-related advantage was observed in women but not in men. Asthma control, when analysed as a continuous score, was better in patients aged ≥75 years compared with those aged 45–64 years. However, when AIRQ scores were collapsed into categorical control levels, no significant differences emerged across age groups. PREMs revealed high satisfaction across all age groups; interpersonal aspects of care–such as respect, autonomy, and communication–were rated more favourably than organisational aspects, such as waiting times and provider choice. Organisational experience improved with age, with patients aged 65–74 years rating it more positively compared to those aged 45–64 years; this age effect was pronounced among women.

Taken together, the observed differences explained only a small proportion of the variance in PROMs and PREMs, indicating that age-related differences in patient-reported quality of care are modest in magnitude. The most consistent pattern was that younger and middle-aged adults, particularly women, reported slightly lower generic HRQoL and less favourable organisational experiences than 65–74-year-olds, whereas categorical asthma control and interpersonal experiences of care were broadly comparable across age groups. These findings therefore point to subtle, descriptive age-related patterns rather than independent age effects. In interpreting these patterns, it should be considered that the digital study design and the relatively small oldest-age stratum may have favored healthier older respondents, and that unmeasured clinical and sociodemographic factors could contribute to the observed differences.

Prior evidence on age-related differences in HRQoL among asthma patients is heterogeneous. A Dutch cross-sectional primary care study found that younger age was associated with poorer disease-specific HRQoL^36^. By contrast, a Dutch two-year longitudinal cohort reported that older age was associated with a decline in HRQoL over time^16^, and that this decline was only weakly related to changes in lung function, suggesting that HRQoL and pulmonary function capture partly distinct aspects of disease burden. German data indicate that older age is negatively associated with the physical component of generic HRQoL, but less so with the mental component, among adult asthma patients^15^. Such discrepancies likely reflect differences in measurement instruments, sampling frames, and whether physical and cognitive domains are aggregated or analysed separately. Consistent with this pattern, Nordic research across three countries has shown that older adults report lower physical but better mental HRQoL scores^18^; a distinction that was not made in the present analysis. Findings from the recent PaRIS study^37^ of adults aged 45 years and older with chronic conditions likewise suggests that older age groups (>75 years) tend to report poorer physical functioning and social participation, whereas overall well-being and mental health remain comparatively preserved. However, PaRIS is not asthma-specific. Against this background, the higher PROPr scores observed in older adults in the present study are compatible with relatively preserved or even better subjective well-being despite age-related physical limitations, although the composite nature of PROPr does not allow separate assessment of physical and mental domains. In the literature, such patterns are commonly discussed under the concept of the “paradox of well-being,” which refers to the relative stability of life satisfaction and subjective well-being in older adulthood despite declining physical health^38,39^. Research suggests that certain factors are associated with higher self-reported health in older adults, including lower levels of multimorbidity, female sex, higher functional and psychological resilience, and greater life-space mobility^39^. Evidence from the COVID-19 pandemic further supports this concept, showing that older adults often maintained stable mental health and subjective well-being under conditions of heightened vulnerability and social restriction^40,41^.

When examining gender subgroups, the results for women aligned with the study’s overall findings. Again, older women (65–74 years) reported better general HRQoL than younger women (18–44 years and 45–65 years). The reasons for age-related differences among women remain uncertain, yet observational evidence links peri- and post-menopausal status to differences in asthma phenotypes, symptom burden, and exacerbation risk, plausibly via hormonal pathways that affect airway inflammation and responsiveness^42–45^. As menopausal status and hormone therapy were not measured, residual confounding cannot be excluded. In addition, cohort- and gender-specific differences in expectations and response styles may contribute: older women may rate their health and care more positively at a given level of clinical burden than younger women, as suggested by findings of the PaRIS^37^ study on self-rated health and well-being. Such expectation- and response-related mechanisms could not be examined with the available data, but provide a complementary explanation to purely biological pathways and underline that age-related differences in PROMs likely reflect both clinical and contextual factors.

Our findings indicate that a substantial proportion of patients (more than 40%) across all age groups achieved well-controlled asthma according to AIRQ, with older adults showing the highest levels of control and the lowest prevalence of very poorly controlled asthma. However, approximately half of patients under the age of 75 reported their asthma as not well controlled or very poorly controlled. Contrary to our findings, a recent German survey from 2024 found that adults aged 60+ had the highest symptom burden and daily impairments, suggesting poorer control than younger adults, with 61% of respondents overall classified as having inadequate asthma control. However, this study could not verify the presence of asthma, and misclassification between asthma and COPD by respondents, especially among older people, cannot be excluded^46^. Direct comparison with this study is therefore limited, as they did not use PROMs, which formed the basis of our analysis. The same applies to a German survey based on 2010 data, which reported that less than 5% of adults with asthma achieved controlled asthma, while two-thirds experienced daily or intermittent limitation^9^.

The results suggest that although mean AIRQ scores differed between age groups, no significant differences in asthma control levels emerged, despite these levels being derived from the AIRQ itself. This pattern is consistent with evidence that the AIRQ was primarily developed and validated for use as a composite control measure with predefined control categories, which have been shown to predict future exacerbations. Given this complexity, the literature supports the use of categorical AIRQ control levels because of their clinical relevance, rather than relying solely on the continuous score^47^. At the same time, the present findings indicate that modelling AIRQ as a continuous score may reveal small between-group nuances that are not reflected in categorical control levels. Taken together, this reinforces the use of AIRQ control classifications for clinical interpretability^47,48^, while acknowledging that continuous AIRQ scores can still be analytically informative for detecting subtle variations in symptom profiles. Given the small effect size and the absence of statistically significant differences in categorical asthma control, the more favourable continuous AIRQ scores in the oldest group are best interpreted as indicating slightly better symptom profiles rather than substantively different asthma control status in routine care.

Asthma patients mostly rated their experience with the healthcare system as good/very good, with the interpersonal domain receiving higher ratings than the organisational domain. This pattern aligns with findings from the general population reported in a German study using a similar PREM Responsiveness^12^. While previous research in the general population has suggested that health system responsiveness ratings tend to improve with age^12^ and that younger age groups are more critical than older groups^49^, this analysis did not reveal a statistically significant association between age groups and overall patient-reported experience among asthma patients. Nonetheless, older patients (65–74 years), especially women, rated their organisational experience more positively than younger patients (18–44 years and 45–64 years), which is consistent with the age-related trend described previously. However, it should be noted that the earlier study included only participants with private health insurance, which may limit comparability^12^. The uniformly high PREM scores observed in the present study also suggest potential ceiling effects and socially desirable responding^50^, particularly in the interpersonal domain, which may obscure more nuanced dissatisfaction with specific organisational aspects such as waiting times or access pathways. Younger adults, who reported somewhat less favourable organisational experiences, may have higher expectations regarding flexibility, digital access, and coordination of care, whereas older adults may be more accepting of organisational constraints. More sensitive or domain-specific PREMs, or qualitative studies, may be required to disentangle these patterns. To the best of the authors’ knowledge, this is the first study to investigate differences in PREMs in patients with asthma across different age groups in Germany. As a result, there is no existing comparative literature to which we can directly refer. This lack of prior research highlights the novelty and importance of these findings, emphasising the need for further studies to validate and extend the results.

From a primary care perspective, the pattern of slightly lower HRQoL and less favourable organisational experience among younger and middle-aged adults, contrasted with broadly comparable categorical asthma control and uniformly high interpersonal ratings, suggests a potential “hidden burden” in younger patients^51^. For these age groups, integrating PROMs and PREMs into routine care may help to identify unmet needs that are not captured by spirometry or categorical control alone, including young patients’ limited perceived benefit from preventive care, insufficient disease-specific education and support, the tendency to normalise chronic symptoms, and competing demands that make asthma management a lower priority in daily life^52^. Conversely, the favourable PROM and PREM profiles in older adults should not be interpreted as implying low need for care; clinicians should remain alert to under-reporting of symptoms, adaptation to longstanding limitations, and the risk that digitally less engaged older patients – who were underrepresented in this sample – may have very different experiences.

### Strengths and limitations

The study has several strengths and limitations that must be acknowledged. It is one of the first to assess PROMs and PREMs simultaneously in patients with asthma. One notable strength is the complete availability of age as a variable, allowing more robust analyses of age-related differences. A limitation is that the sample size varied between age groups. In particular, the ≥75-year group was comparatively small, which limits precision and statistical power for detecting age-group differences and increases uncertainty of estimates for this stratum. This may also have affected the robustness of between-group comparisons^53,54^. In addition, the effect sizes for age-related differences in PROMs and PREMs were consistently small, limiting the clinical relevance of group differences at the individual level. However, these differences may still be informative for population-level monitoring and benchmarking.

Furthermore, the representativeness of the results may be limited as the digital nature of the study tends to underrepresent certain population groups^55^. All participants were insured with a single statutory health insurer (BARMER), and women constituted around two-thirds of the sample across age groups. This female predominance is broadly compatible with the higher asthma prevalence and severity observed among adult women in Germany and internationally^1,25^, but it may still limit generalisability to male patients and to individuals insured with other sickness funds or private insurance. As no adjustment was made for potential confounders, the possibility of confounding cannot be excluded^56^. Important unmeasured or uncontrolled variables include comorbidities (e.g., depression, obesity), smoking status, asthma severity and endotype, DMP enrolment status, and menopausal or hormonal status in women, all of which may influence both clinical status and patient-reported outcomes. In addition, PROMchronic analyses of survey adherence indicate lower participation at older ages, with the lowest initiation and implementation among patients aged ≥75 years. Overall, male sex was negatively associated with initiation; however, men aged ≥75 years were more likely to initiate than expected from the main effects of age and gender. *Initiation* refers to enrolling/starting the survey process (i.e., providing baseline participation), whereas *implementation* refers to completing at least one electronically collected questionnaire after enrolment. Moreover, the negative age-gradient in initiation was reported to be more pronounced among women, while lower or non-disclosed household income was associated with lower implementation, suggesting underrepresentation of older and socioeconomically disadvantaged groups in digitally collected ePROM/ePREM data^55^. This selection may lead to overestimation of HRQoL and organisational experiences in the older asthma population.

PREMs reflected the most recent outpatient consultation with either a general practitioner or specialist. If the distribution of provider types differs by age group, this could confound patient-reported experiences, as expectations and interactions may vary between GPs and specialists. Provider type was not analysed in detail in this manuscript and therefore remains a potential source of residual confounding.

Despite using repeated claims-based ICD-10-GM codes and applying the M2B criterion, misclassification between asthma and COPD, particularly in older adults, cannot be fully ruled out due to their clinical overlap and the absence of clinical validation data.

The chosen age groups are a limitation, as they may not fully capture the complexity of age-related differences. The varying width of categories may mask significant differences within broader bands, meaning the groups may not accurately reflect all changes in health and experience between them^57^.

Future research may benefit from a more refined age categorisation, particularly by further breaking down the 18–44 years group^57^, provided sufficient sample size per group. It would also be valuable to investigate whether participation in DMPs is associated with improved patient-reported quality of care in specific age groups. As these programmes are designed to provide structured care for patients with asthma, their effectiveness may vary by the age of enrolled patients. Furthermore, differentiating by asthma endotypes could reveal whether certain patient groups perceive differences in quality of care, as different endotypes require specific treatment approaches^58^ that may influence PROMs and PREMs across age groups. A more detailed analysis of these factors could provide insights into age-related differences in patient-reported quality of care in Germany and help identify which aspects of care require further optimisation.

## Conclusions

This study indicates small, domain-specific age differences in patient-reported quality of care among adults with asthma. Older adults, particularly women aged 65–74 years, reported higher generic HRQoL and better asthma control on the continuous scale at ≥75 years, while asthma control levels and most PREM domains showed no significant differences. Within this digitally engaged population insured with a large statutory health insurer, age was therefore associated with subtle shifts in how asthma patients perceive their health and organisational aspects of care. In contrast, interpersonal experiences of care appeared consistently positive across age groups.

These findings suggest that age may shape patients’ perceptions of care, underlining the potential value of age-segmented reporting and targeted strategies to optimise patient engagement and care delivery. In particular, PROMs and PREMs may help to identify a burden among younger and middle-aged adults whose clinical asthma control appears similar to that of older patients, but who report slightly lower HRQoL and less favourable organisational experiences. Future research should explore underlying mechanisms and examine how longitudinal assessments of PROMs and PREMs can inform interventions to improve quality of care across diverse adult age groups.

### Ethics approval and consent to participate

This study was conducted in accordance with the Declaration of Helsinki. The PROMchronic study was approved by the Charité’s Ethics Committee, Berlin (Date: 21.08.2023, No: EA2/035/23) and registered with the German Register for Clinical Studies (DRKS) under DRKS00031656. Each patient gave informed consent to participate in the study.

## Supplementary information

Supplementary Information

## Data Availability

The datasets used and analysed in the current study are not publicly available under German data protection law, but are available from the corresponding author upon reasonable request.

## Code availability

The underlying code for this study is not publicly available but may be made available to qualified researchers on reasonable request from the corresponding author.
