# Supplementary material for: Age-related differences in patient-reported quality of care among adult German patients with bronchial asthma: a cross-sectional study
Source: NPJ Prim Care Respir Med. 2026 Feb 23;36:16. doi: 10.1038/s41533-026-00492-8 (PMC12993078; doi:10.1038/s41533-026-00492-8)
Supplement: Supplementary file 1 — Supplementary Information [file 41533_2026_492_MOESM1_ESM.docx]

# Age-related differences in patient-reported quality of care among adult German patients with bronchial asthma: a cross-sectional study – Supplementary Information

Supplementary Table 1: Summary statistics for PROPr and AIRQ stratified by age and gender

| **PROM^1^** | **18-44 years** | **45-64 years** | **65-74 years** | **≥ 75 years** | **Test (Statistic, p, Effect size)** |
| --- | --- | --- | --- | --- | --- |
| **Women** | (n = 98) | (n = 344) | (n = 82) | (n = 32) |  |
| **PROPr** |  |  |  |  | ANOVA, F(3, 552) = 3.622, p = 0.013, η² = 0.019  65-74 vs. 18-44 Tukey’s test, p = 0.032, Cohen’s d = -0.415  65-74 vs. 45-64 Tukey’s test, p = 0.012, Cohen’s d = -0.375 |
| Mean (SD) | 0.36 (0.20) | 0.36 (0.21) | 0.44 (0.20) | 0.40 (0.20) |  |
| Median (IQR) | 0.35  (0.189 - 0.501) | 0.34  (0.205 - 0.496) | 0.43  (0.321 - 0.556) | 0.39  (0.292 - 0.52) |  |
| **AIRQ** |  |  |  |  | Kruskal-Wallis test, χ^2^(3) = 8.042, p = 0.045, η² = 0.009 |
| Mean (SD) | 2.26 (2.33) | 2.74 (2.46) | 2.45 (2.26) | 1.75 (2.29) |  |
| Median (IQR) | 2 (0-4) | 2 (0-4) | 2 (0.25-4) | 1 (0-2) |  |
| **Asthma control** n (%) |  |  |  |  | Fisher’s exact test, p = 0.164, Cramér’s V = 0.074 |
| Well-controlled | 47 (48.0) | 139 (40.4) | 36 (43.9) | 17 (53.1) |  |
| Not well-controlled | 34 (34.7) | 122 (35.5) | 29 (35.4) | 12 (37.5) |  |
| Very poorly controlled | 17 (17.4) | 83 (24.1) | 12 (20.7) | 3 (9.4) |  |
| **Men** | (n = 35) | (n = 102) | (n = 53) | (n = 17) |  |
| **PROPr** |  |  |  |  | Kruskal-Wallis test, χ^2^(3) = 1.623, p = 0.654, η² = 0 |
| Mean (SD) | 0.46 (0.20) | 0.44 (0.22) | 0.47 (0.24) | 0.49 (0.17) |  |
| Median (IQR) | 0.43  (0.293 - 0.608) | 0.44  (0.254 - 0.575) | 0.50  (0.290 - 0.640) | 0.50  (0.352 - 0.598) |  |
| **AIRQ** |  |  |  |  | Kruskal-Wallis test, χ^2^(3) = 5.763, p = 0.124, η² = 0.014 |
| Mean (SD) | 2.29 (2.04) | 2.37 (2.53) | 1.74 (2.41) | 1.35 (2.06) |  |
| Median (IQR) | 2 (0-4) | 2 (0-4) | 0 (0-3) | 0 (0-2) |  |
| **Asthma control** n (%) |  |  |  |  | Fisher’s exact test, p = 0.142, Cramér’s V = 0.158 |
| Well-controlled | 14 (40.0) | 46 (45.1) | 32 (60.4) | 12 (70.6) |  |
| Not well-controlled | 16 (45.7) | 33 (32.4) | 15 (28.3) | 3 (17.7) |  |
| Very poorly controlled | 5 (14.3) | 23 (22.6) | 6 (11.3) | 2 (11.8) |  |

^1^The score ranges for the PROMs are -0.022 to 1 for the PROPr, with higher scores indicating better health, and 0 to 10 for the AIRQ, with higher scores indicating poorer asthma control (0 to 1: Well controlled, 2 to 4: Not well controlled, 5 to 10: Very poorly controlled). Analyses were restricted to men and women due to low counts in the ‘diverse’ category (2 participants excluded: 18-44 and 45-64).

Abbreviations: PROM = Patient-reported outcome measure, ANOVA = Analysis of Variance, PROPr = PROMIS-Preference Score, SD = standard deviation, IQR = interquartile range

Supplementary Table 2: Summary statistics for PREM Responsiveness overall and organisational and interpersonal domains stratified by age and gender

| **PREM^1^** | **18-44 years** | | **45-64 years** | | **65-74 years** | | **≥ 75 years** | **Test (Statistic, p, Effect size)** |
| --- | --- | --- | --- | --- | --- | --- | --- | --- |
| **Women** | (n = 98) | | (n = 344) | | (n = 82) | | (n = 32) |  |
| **Responsiveness** n (%) |  | |  | |  | |  | Fisher’s exact test, p = 0.164, Cramér’s V = 0.095 |
| Moderate/bad/very bad | 11 (11.2) | | 52 (15.1) | | 5 (6.2) | | 4 (12.5) |  |
| Very good/good | 87 (88.8) | | 292 (84.9) | | 76 (93.8) | | 28 (87.5) |  |
| Not applicable | 0 | | 0 | | 1 | | 0 |  |
| **Responsiveness – Organisational domain** n (%) |  | |  | |  | |  | Chi-squared test, χ^2^(3) = 11.684, p = 0.009, Cramér’s V = 0.145  18-44 vs. 65-74 Chi-squared test, χ^2^(1) = 8.074, p = 0.027, Phi = 0.208  45-64 vs. 65-74 Chi-squared test, χ^2^(1) = 12.438, p = 0.006, Phi = 0.161 |
| Moderate/bad/very bad | 29 (29.6) | | 105 (30.5) | | 10 (12.4) | | 7 (21.9) |  |
| Very good/good | 69 (70.4) | | 239 (69.5) | | 71 (87.7) | | 25 (78.1) |  |
| Not applicable | 0 | | 0 | | 1 | | 0 |  |
| **Responsiveness – Interpersonal domain** n (%) |  | |  | |  | |  | Fisher’s exact test, p = 0.837, Cramér’s V = 0.042 |
| Moderate/bad/very bad | 7 (7.1) | | 34 (9.9) | | 6 (7.4) | | 3 (9.4) |  |
| Very good/good | 91 (92.9) | | 310 (90.1) | | 75 (92.6) | | 29 (90.6) |  |
| Not applicable | 0 | | 0 | | 1 | | 0 |  |
| **Men** | (n = 35) | | (n = 102) | | (n = 53) | | (n = 17) |  |
| **Responsiveness** n (%) |  | |  | |  | |  | Fisher’s exact test, p = 1, Cramér’s V = 0.032 |
| Moderate/bad/very bad | 3 (8.6) | | 9 (8.8) | | 5 (9.4) | | 1 (5.9) |  |
| Very good/good | 32 (91.4) | | 93 (91.2) | | 48 (90.6) | | 16 (94.1) |  |
| Not applicable | 0 | | 0 | | 0 | | 0 |  |
| **Responsiveness – Organisational domain** n (%) |  |  | |  | |  | | Fisher’s exact test, p = 0.923, Cramér’s V = 0.055 |
| Moderate/bad/very bad | 9 (25.7) | 27 (26.5) | | 14 (26.4) | | 3 (17.7) | |  |
| Very good/good | 26 (74.3) | 75 (73.5) | | 39 (73.6) | | 14 (82.4) | |  |
| Not applicable | 0 | 0 | | 0 | | 0 | |  |
| **Responsiveness – Interpersonal domain** n (%) |  |  | |  | |  | | Fisher’s exact test, p = 1, Cramér’s V = 0.024 |
| Moderate/bad/very bad | 2 (5.7) | 7 (6.9) | | 3 (5.7) | | 1 (5.9) | |  |
| Very good/good | 33 (94.3) | 95 (93.1) | | 50 (94.3) | | 16 (94.1) | |  |
| Not applicable | 0 | 0 | | 0 | | 0 | |  |

^1^PREM responses were dichotomized into 'very good/good' and 'moderate/bad/very bad’. Analyses were restricted to men and women due to low counts in the ‘diverse’ category (2 participants excluded: 18-44 and 45-64).

Abbreviations: PREM = Patient-reported experience measure
